# The use of a digital life story to support person-centred care of older adults with dementia: A scoping review

**DOI:** 10.1177/20552076241241231

**Published:** 2024-03-20

**Authors:** Helén Dellkvist, Ana Luiza Dallora, Line Christiansen, Lisa Skär

**Affiliations:** 1 Department of Health, 4206 Blekinge Institute of Technology, Karlskrona, Sweden; 2 Faculty of Health Science, 4342 Kristianstad University, Kristianstad, Sweden

**Keywords:** Digital life story, life story, person-centred care, residential care, scoping review

## Abstract

**Introduction:**

A life story (LS) is a tool healthcare professionals (HCPs) use to help older adults with dementia preserve their identities by sharing their stories. Applied health technology can be considered a niche within welfare technology. Combining technology and nursing, such as using life stories in digital form, may support person-centred care and allow HCPs to see the person behind the disease.

**Objective:**

The study's objective was to summarise and describe the use of life stories in digital form in the daily care of older adults with dementia.

**Methods:**

A scoping review was conducted in five stages. Database searches were conducted in Cinahl, PubMed, Scopus, Web of Science, and Google Scholar; 31 articles were included. A conventional qualitative content analysis of the collected data was conducted.

**Results:**

The qualitative analysis resulted in three categories: (1) benefits for older adults, (2) influence on HCPs’ work, and (3) obstacles to implementing a digital LS in daily care.

**Conclusion:**

Older adults with dementia can receive person-centred care through a digital LS based on their wishes. A digital LS can enable symmetric communication and serve as an intergenerational communication tool. It can be used to handle behavioural symptoms. Using a digital LS in the later stages of dementia may differ from using it earlier in dementia. However, it may compensate for weakening abilities in older adults by enhancing social interaction.

## Introduction

Older adults with dementia may struggle to express their needs and wishes due to communication difficulties and memory loss.^1^ In person-centred care (PCC), it is imperative to know the person, and life story (LS) work is one tool used to accomplish this in daily care by helping older adults preserve their identities through sharing their stories.^2^ According to Alzheimer's Disease International,^3^ every 3 s, someone in the world develops dementia. Over 55 million people are living with dementia worldwide, and in 2050, this number is estimated to reach 139 million. The expected increase is mainly due to the population living longer and healthier, with a more significant proportion of older adults.^3^ In Sweden, many older adults with dementia live in residential care settings, which, according to the definition by the National Board of Health and Welfare in Sweden,^4^ refers to individual assisted accommodation for older adults 65 years and older. This accommodation is provided with the support of the Social Services Act or the Act on Support and Services for certain disabled persons.^4^ In this study, a healthcare professional (HCP) is a specialist, registered, or enrolled nurse who provides care and nursing to residents in residential care. Although relatives are important, this study focuses on the perspective of HCPs.

An LS describes who the person is. It shapes the person, and the person shapes their LS. It makes sense that one's life helps shape one's story and allows one's feelings and experiences to be shared.^5^ An LS is often created by the close relatives of older adults with dementia by completing a document with different designs. The document contains areas where it is possible to describe the older adult from childhood onwards. The older adult's needs, wishes, joys, and sorrows through life can also be added to the document.^6^ These documents are often in written form, and close relatives describe them as extensive, rigid, and complex to complete.^7^

Previous research has shown that an LS is intended as a working tool for HCPs to create security and facilitate communication with older adults with dementia. Research has demonstrated the possibility of supporting PCC using an LS designed based on the individual's needs and wishes.^3^ However, to make the LS usable, HCPs must involve close relatives in contributing valuable information while preserving the identity and dignity of older adults with dementia. Moderate to severe dementia is often characterised by long-term care relationships. The older adult must feel cared for and understood regarding what is essential. Eley and Kaiser^8^ believe that a written LS with personal information, photos, and personal objects of importance can contribute to preserving the older adult's identity. Through HCPs’ active use of the LS, the person ‘behind’ the dementia disease can be seen and heard, and PCC can be supported.^9^ LS work may allow older adults with dementia to endure making and remaking their identities, thus assisting them in communicating aspects of themselves in a safe environment.^9–11^ As stated by Karlsson et al.,^12^ through an LS, it may be possible for one to keep doing things they used to do in the past, which may lead to personal growth throughout their life course. An LS should include past, present, and future and can only be a living document if updated and added to over time. It is only valuable if used to provide PCC.^13^ Creating an older adult's LS is an ongoing process that must be easily accessible to those directly affected and easy to use and update.

Even though HCPs in residential care strive to provide PCC to older adults with dementia, there is a risk that they will mainly see the disease instead of an older adult with a past, present and future. In such situations, a DLS can be an easily accessible tool for HCPs to care for older adults with dementia.^14^ Knowing about an older adult's past may lead to understanding what matters in their present.^15^ A DLS can address the problems through technology and nursing, such as in applied health technology, which can be considered a niche within welfare technology. Welfare technology is defined by the National Board of Health and Welfare in Sweden^16^ as ‘digital technology that aims to maintain or increase the safety, activity, participation or independence of a person who has or is at increased risk of having a disability’. Technologies have become increasingly implicated in caring for older adults. However, designs for care settings may face difficulties when used in real-life contexts. Nursing, which contains soft values, must be combined with digitalisation, and to enable this, older adults must be allowed to be co-creators, users, and recipients of technological innovations.^17^ Manchester and Jarke^18^ emphasise the importance of co-designing with HCPs to find openings for technology to be a natural and appreciated part of daily care, which may be a complex intervention to implement. As stated by Frennert,^19^ using technology in daily care is an intervention that may bring both potential and friction. A DLS can be used in different forms. It can be assisted by several software programs and applications, which may provide various sources for collected content and personal and generic material.^20^ If the LS is digital, it may be accessible for HCPs to provide PCC. In the research field, reviews regarding the DLS exist, but mainly from other perspectives, such as older adults’ or relatives’ perspectives. Hence, this scoping review aims to summarise and describe the advantages and disadvantages for HCPs of using a DLS in caring for older adults with dementia in daily care. The review provides a foundation for future studies regarding opportunities for the abovementioned objective.

## Methods

### Design

A scoping review, with five stages described by Arksey and O’Malley,^21^ was chosen because it is an appropriate way to obtain a quick overview of the extent of a research area without necessarily obtaining detailed research findings. In Stage 1, the research question was identified: How is the LS in a digital form used worldwide when caring for older adults with dementia, and can it support PCC? The Preferred Reporting Items for Systematic Reviews and Meta-Analyses extension for scoping reviews (PRISMA ScR)^22^ was used to present the results of the included articles and increase methodological transparency. The protocol for this scoping review was not registered and publicly available, but it can be provided by the corresponding author upon request. Since this is a scoping review without study participants, no ethical approval or informed consent was necessary.

### Data search and selection

**Figure 1.**
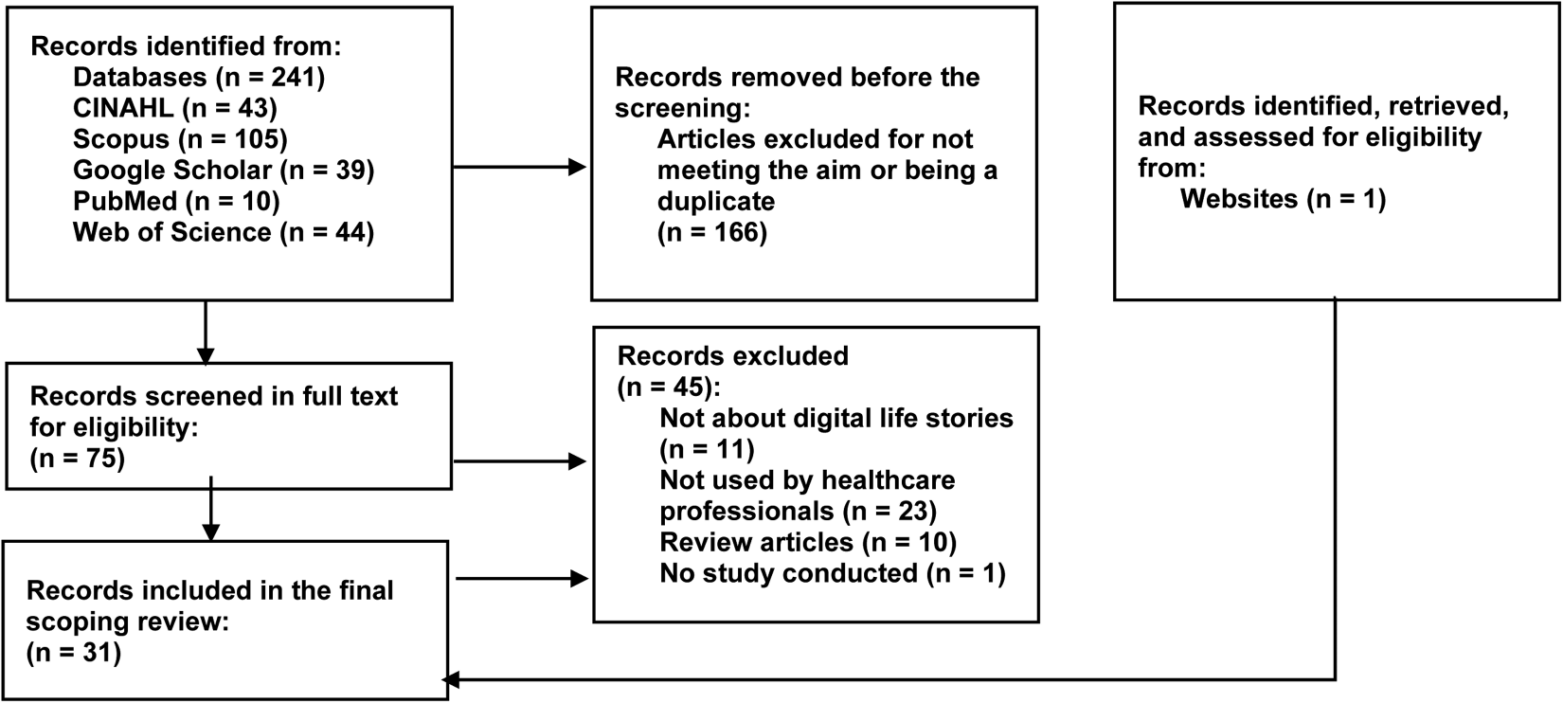
Flowchart of the search process.

In Stage 2, relevant studies meeting the aim were identified. The research question laid the foundation for the data search. Before database searches began, a librarian was consulted, as suggested by Arksey and O’Malley,^21^ to identify relevant databases and search terms. The CINAHL, Scopus, Google Scholar, PubMed, and Web of Science databases were chosen to cover health, nursing, and engineering research. Before the final search terms were determined, the authors discussed alternative terms. When searching PubMed, the term ‘multimedia technology’ was added. Initially, we chose to broaden our search without using ‘HCPs’ since narrowing it could lead to a risk of missing out on relevant material. It was only in Google Scholar that the search term ‘HCPs’ was added since we wanted to narrow our search. In Scopus, the following limitations were added: social sciences as a field of study and the keywords ‘autobiography’ and ‘life history’. These abovementioned changes narrowed the search to meet the aim. The final search terms are presented in Appendix 1, and the database searches are shown in Appendix 2. Supplementary articles were identified through reference lists and searched for separately in named databases. A web-based presentation was found when searching the internet for material related to an article about an application. Both database and supplementary searches were completed in February 2023. In Stage 3, the scoping process began. The eligibility criteria for this scoping review were studies in English, including those on older adults (65 and older) living with dementia, HCPs, DLSs, and DLS work in other formats. The concepts that should be present in the included material were LS, PCC, residential care, and DLS. The review covers studies conducted in residential or home care facilities. Review articles and articles about storytelling and informal caregivers were excluded. No limitation was placed on the publication date since we strived for all accessible data. Regarding the search results, titles and abstracts were first screened to identify relevant articles. Full-text articles were read when they seemed suitable for this scoping review. This screening was performed by the first author (HD) and was discussed with the other authors. Figure 1 presents a flowchart of the search process inspired by a PRISMA 2020 flow diagram.^23^

### Data summary and analysis

Stage 4 involved charting the data to obtain an overview (see Appendix 3). An overview of the included articles is shown in Appendix 3 for reference. The included articles were analysed through qualitative content analysis on the studies’ full text, according to Hsieh and Shannon's^24^ method. The first author was primarily responsible for conducting the analysis, and the process was continuously presented to and discussed with the other authors. Shared reflections permeated the development of categories and the presented results. According to Hsieh and Shannon's^24^ description of conventional qualitative content analysis, Stage 5 is the time to collate, summarise and report the results. All data were read repeatedly to achieve immersion and a sense of the content as an entity. Data were read word by word to obtain codes and capture key thoughts and concepts. The data were approached by making notes of the first impressions and ideas as an initial analysis. The labels for codes reflecting more than one key thought emerged directly from the data and became the initial coding scheme. After that, codes were sorted into subcategories, and finally, subcategories were combined, based on their relationships, and organised into categories. A diagram was used to manage the categories into a structure.

## Results

### Overview

Thirty-one studies published between 2004 and 2021 were included (see Appendix 3). These included research articles (17/31),^25–41^ conference articles (8/31),^42–49^ theses (2/31),^50,51^ discussion articles (1/31),^52^ long papers (1/31),^53^ ministry reports (1/31),^54^ and a web-based presentation (1/31).^55^ The included material originated from the United Kingdom (12/31),^25,26,31,33,41,43,45,47,52–55^ Sweden (5/31),^32,38,40,50,51^ Australia (3/31),^36,39,48^ the United States (2/31),^28,30^ Canada (2/31),^27,35^ the Netherlands (1/31),^29^ Austria (1/31),^42^ Finland (1/31),^37^ Denmark (1/31),^34^ Malaysia (1/31),^44^ Spain (1/31),^56^ and Hong Kong (1/31).^46^ Both qualitative and quantitative designs were used in the included material. Several different forms of a DLS emerged, such as using computers (USB), iPhones, iPads, interactive artwork, and DVD movies. The summarised results are presented in the following categories: (1) *benefits for older adults*, (2) *influence on HCPs’ work*, and (3) *obstacles to implementing a DLS in daily care.*

### Benefits for older adults

DLSs could support PCC for older adults with dementia by enhancing reminiscence, communication and interaction with HCPs and relatives, evidenced by HCPs’ enjoyment of listening to older adults telling their stories. A finding was that older adults with dementia could use touch screens and laptops to share memories and interact regarding personal content, such as photos from childhood, holidays, marriage, family and friends, videos, and music to evoke memories and emotions.^25–35,42–44,50–54^ Multimedia included meaningful content that appealed to cognitive processes such as communication, recognition, viewing imagery, and hearing music.^27^ This could provide a good atmosphere where DLSs enable older adults with dementia to participate more actively and equally in conversations with HCPs, relatives and friends.,^25–35,42–45,50–54^ reduced the pressure to answer questions and increased self-esteem.^26–29,42,50^ Another positive effect of the DLS was that relatives and friends visited more frequently.^36^ DLSs could also help improve quality of life, making older adults with dementia proud of their lives.^33,37,44^ DLSs might assist older adults with dementia maintain cognitive processes and preserve a sense of self and familiarity, where local references, culture and nature appear to be potential memory triggers.^27^

Through technology, the care environment could become a place of opportunity where the younger and older generations are connected through their device experiences. The DLS also provided an external memory aid to assist procedural memory, such as choosing what to eat. The ease with which an LS could be recorded was also noted.^54^ Remembering things from the past seemed to be more accessible than talking about new things.^32^ Music and photos seemed easier for older adults to understand than text material, which required more prompting. On several occasions, when older adults shared memories, they directly responded to prompts provided by the DLS, such as hearing a long-loved song or seeing a familiar name or face, which seemed to enhance reconnection to a younger self.^30,46^

### Influence on HCPs’ work

DLSs supported contact and communication for HCPs and older adults, especially for older adults with communication problems due to disease.^37^ They influenced HCPs by facilitating discussions with older adults based on their past and present interests.^47^ The digital aspect was a learning experience for HCPs. They became more confident as the sessions continued.^31^ The devices were perceived as supporting older adults in engaging in occupations aligned with their interests and LSs.^56^ HCPs connected with older adults in new ways through verbal and non-verbal responses, and over time, increasing and symmetric two-way communication allowed older adults to make more choices and take more initiative. The older adults expressed enjoyment, smiling, laughing, singing, moving to music, and commenting on specific people and relationships.^25–28,30,32,35–37,42,47,50–52^ The HCPs suggested more use areas for DLSs, such as in team meetings to discuss observed behavioural symptoms,^52^ training new employees,^34,38,47^ communicating with relatives,^31^ and for intergenerational communication, described as a way to transmit family history.^30,31,33,37,39,54,55^

DLSs were valuable and beneficial in PCC for supporting older adults’ privacy, dignity, and memories, especially in the early stages of dementia when the older adult might have more maintained functions and could participate in activities using the DLS.^36,48^ DLSs changed HCPs’ views of older adults and helped them recognise and prevent disruptive behaviour, which relieved their burden and the older adult's distress.^40,41,47,54^ The HCPs needed help from relatives to compile and update content for the DLSs.^34^ They shared goals and experiences with relatives on a shared platform with multimedia content.^29,38^

The HCPs enjoyed using and updating the DLSs regularly and accessed more material from a repository.^30,31,37,44,52^ The DLSs enabled planning and supporting daily activities aligned with older adults’ interests.^27,29,33,47,49,51^ The HCPs liked the diversity, choice, and ease of interface, and the technology helped them think creatively and learn more about older adults, and the DLSs offered possibilities for caring for older adults with dementia by sharing novel content and memories. Sometimes, the older adults with dementia recalled memories and facts about their history that no one had heard.^25,32,42,47,50,52,53^ The DLSs contained various materials to provide diversity, engagement,^28,29,41,42,46^ and calmness for older adults with dementia, for example, when exhibiting agitation or other challenging behaviours.^31,33,46–48^ Another benefit for HCPs might be that having a record of activities and their responses on a DLS could enable them to keep track of decline between periods.^38^

Using DLSs allowed HCPs to add names to photos for clarification purposes.^37^ The personalised ways in which HCPs could use the devices enabled them to get to know the older adults as persons, which helped HCPs discover their interests and capacities. The HCPs had favourable views of the technology and DLS's impact on them and their clients. The technology offered many resources for discussion,^54^ and HCPs rated their engagement with DLSs higher than with LSs in written form.^39^ The technology could provide more effective support for HCPs than older adults whose interactions with mobile devices are often limited due to physical and cognitive impairment.^42^ Initially, DLSs evoked mixed emotions in older adults when looking at old photos.^30,32,33,37,54^ and HCPs focused on photos of older adults, as photos of relatives could confuse them.^36^ Recent photos were less engaging, and DLSs should be used carefully and not too long. The level of engagement depended on daily conditions and medication.^36,47^

### Obstacles to implementing a DLS in daily care

Obstacles to DLS implementation for older adults with dementia were identified. The administrator's role required time for training and time to use and add material to the DLS.^27,43,46,54,56^ HCPs’ attitudes, values, and confidence in using technology varied. The HCPs differed in using DLSs and were unsure about access, number, and implementation of the DLSs.^34,36,40,52,54^ DLS and technology use required flexible care settings and training. HCPs faced stress due to time constraints and the need to align the DLS with the prevailing care routines to motivate its use as an integrated aspect of care. The HCPs also needed care setting managers to do more to gain approval for using the DLS.^34,36,52^

The HCPs mentioned obstacles, including problems logging in and out of the DLSs, issues recording a reaction, accidentally resetting the application, and other technical problems that might arise. The content within the DLS must be easily adaptable after the initial set-up to account for changing needs with disease progression and unpredictable responses to media.^36^ The application was not always user-friendly, with sensory, aural, and visual obstacles. Some older adults had physical barriers to DLS use and device management due to the device weight and screen size.^44^ Privacy could be an obstacle, as the device was used as a shared resource in the care setting and could be used by multiple users. Therefore, a user login was crucial.^38^ Some concern was also directed at Wi-Fi connectivity and security issues when integrating the touchscreen technology within the care setting, as in who would have access to the content on the device.^54^

## Discussion

The results show different technological tools and various ways to use the LS in digital form to support the PCC of older adults with dementia. The results indicate that a DLS can benefit older adults and influence HCPs’ work, although there may be obstacles to implementation in daily care. Essential aspects that should be considered are that the DLS should be easily accessible and easy to use based on the older adult's needs and wishes.

Regarding benefits for the older adult, the results show that personal multimedia content from the past significantly influences older adults regarding communication, social interaction, and recognition, which can lead to a more equitable conversation with HCPs and relatives. This aligns with previous research^57,58^ finding that stories and photos of close relatives, family holidays, and the older adult's early life are particularly special to them and often lead to meaningful conversations. Notably, all this can be done through a traditional LS. Still, the results indicate that it can be improved with a DLS, for example, by adding videos and music. A DLS is also accessible with all content in one tool and is easy to update. Piasek et al.^57^ found that none of the stories emerged from a more recent period of the older adult's life. Critten and Kucirkova^59^ argue that the strongest memories are from older adults’ youth, and memories from later times appear less significant. The results indicate that using material, such as that from a hometown, familiar sounds, or reading an old recipe provides various forms of engagement and could help calm, relax, and engage older adults with dementia who exhibit agitation and other challenging behaviours. According to Crete-Nishihata et al.,^60^ looking at an old photo on a device can make the older adult remember and almost feel the smell and taste and, in a way, return to where it happened. This is aligned with Goodall et al.'s^61^ and Tolo Heggestad and Slettebö's^8^ findings regarding how individualised digital technologies can positively affect older adults with dementia in terms of their well-being, particularly in behaviour and mood, their sense of identity, social dignity, and relationships with others.

The results also show that music and photos seem more straightforward to understand than text material on the device. This aligns with Sweeney et al.'s^62^ finding that images are essential to a DLS. Photos help cue the parts together, and creating a DLS seems more enjoyable than just looking at a photo. Cooney et al.^14^ found that a DLS facilitates two-way conversation, both verbal and non-verbal, which builds relationships and increases interaction between the older adult and the HCP. Manchester and Jarke^17^ support this finding in that personal objects, smells and sounds from the past can create temporal links between the past and the present, enabling older adults to share stories from their lives and make new connections with others in meaningful ways.

Furthermore, the results highlight that the DLS is seen as a means to compensate for weakening abilities through meaningful daily activities aligned with the older adult's interests and life history. Most HCPs feel that the DLS is something they can use and continue adding to and integrating into daily care, preferably with supportive management. Gridley et al.^3^ state that HCPs appreciate the opportunity to update the DLS regularly, which digital technologies facilitate. However, there are challenges, such as who should be responsible for keeping the DLS current, and Gridley et al.^3^ further argue that one suggestion is to combine the daily reports in the team with work concerning the DLS. Virtanen et al.^63^ indicate that more efforts are needed to increase HCPs’ motivation to use new techniques, and they stress the impact of the social environment, such as encouragement and acceptance of eHealth.

The findings of this study further indicate that technology can aid interaction with close relatives and link younger and older generations through sharing moments in residential care with relatives and transmitting family history, acting as a legacy from the older adult to the coming generations. This is supported by Crete-Nishihata et al.,^60^ Ryan et al.,^58^ and Swan et al.,^56^ who revealed HCPs’ views that the content on iPads facilitates the relationship between older adults and their families to enable conversations about the LS. Close relatives can access the material in the DLS, which gives them insight into life in residential care, and this can motivate them to be engaged in using the DLS during visits. Not all older adults living in residential care have relatives to assist in adding relevant material, which can lead to an unfair situation. Using various parts of the material also provides diversity and elicits varied forms of engagement. According to Swan et al.,^56^ iPads used for the DLS can support older adults in engaging in activities aligned with their interests, for example, seeing a photo of themselves at home as a younger person and sharing stories from that time. McKeown et al.^10^ found that the DLS could be overused in caring for older adults, leaving a feeling of discomfort. Therefore, HCPs need to observe signs of disengagement to avoid pressure on the older adult to participate in using the DLS.

Even though time constraints may occur, the results show that HCPs appreciate the DLS as an enrichment of their work and that they plan on using it regularly. A lack of technical skills and uncertainty about use may hinder engagement in incorporating work with the DLS into daily care. This indicates that training for HCPs might be necessary before introducing a new digital tool. Gridley^3^ also found that understanding the challenges is essential to bringing forward personhood and tailoring daily care to meet the needs of the older adult. Hung et al.^64^ discuss the value of HCPs applying a working-with approach from the older adult's point of view rather than using a doing-to method.

Another finding is that using a DLS can help calm and relax older adults with dementia who exhibit agitation and other challenging behaviours. Cooney et al.^14^ highlight that reminiscing provides HCPs with strategies to help them manage behavioural symptoms solely by knowing the person better. According to Gridley et al.^3^ and Swan et al.,^56^ HCPs describe that a DLS, even in the later stages of dementia, can help in handling challenging behaviours and has the potential to solve problems. Hung et al.^64^ and Ryan et al.^58^ state that the contents of or about nature bring feelings of safety and security, enabling the older adult to relax.

One obstacle to implementing a DLS is that looking at old photos on the DLS can be associated with mixed emotions and, at first, can seem to evoke anguish and sad memories. McKeown et al.,^10^ Goodall et al.,^61^ and Critten and Kucirkova^59^ emphasise that private and intimate disclosures may emerge as an uninvited consequence of looking at photos of loved ones who are not alive and talking with the older adult about their LS; therefore, preserving and maintaining the older adult's privacy and dignity is crucial. Another obstacle is the physical barriers that several older adults face when using a DLS. Critten and Kucirkova^59^ mention how using technology and devices may cause problems due to physical constraints such as poor eyesight and hand coordination.

The results show that research about DLS can provide new awareness, enabling HCPs to offer versatile and tailored care according to what is essential for the older adult. Technology can facilitate the DLS by making it easily accessible, easy to use, and updated. To support its use in residential care settings, management must learn about its advantages and act as facilitators. Time constraints may hinder HCPs from regularly using a DLS in daily care and updating it with the older adult. Frennert^19^ states the importance of reducing technology knowledge and experience imbalance among HCPs and the lack of resources and structures to accomplish a digital implementation, and Manchester and Jarke^18^ discuss the need for including both HCPs and older adults to co-design to make the best out of the product in question. As Kirkevold et al.^65^ and McKeown et al.^10^ state, HCPs may, through the DLS, improve communication with and more easily get to know the person with dementia and their relatives, which leads to increased understanding. Further research is needed to investigate the effects of implementing a DLS in residential care settings, for example, through focus group interviews and observation studies.

## Methodological considerations and limitations

This scoping review focused on a wide rather than in-depth synthesis of available research.^21^ Hence, several databases were used to find as much relevant material as possible. To scope the area for material about the use of DLSs, the authors even included grey literature, such as theses, study protocols, work-in-progress papers, and a web-based presentation when the inclusion criteria were met.^21^ The weight of the results differs between small sample size studies and bigger sample sizes. There is a need for more research in this area, and as there are still few studies, a scoping review was appropriate. However, the purpose of our study was to summarise and describe the use of DLSs in the daily care of older adults with dementia, regardless of the included material's sample size, in this case, from a qualitative perspective. Notably, most of the included material was from the United Kingdom, which indicates a leading position in the field. Publication dates for the included articles ranged from 2004 to 2021, with most studies being conducted in 2016 and 2020. No absolute conclusion can be drawn from the number of studies included since the authors may have missed some existing studies. The first author (HD) performed the search and screening processes with initial support from an experienced librarian. Perhaps relevant material that might have been found if more than one researcher had performed the procedures or if a systematic literature review had been conducted instead was missed. We excluded reviews from the results, as there was a risk of bias in interpreting other researchers’ interpretations, and this supports the current study's credibility. Additionally, a scoping review does not seek to evaluate the impact or quality of the results of the included material.^21^ Thus, no quality evaluation was conducted during the inclusion process. All authors discussed the analysis process to increase trustworthiness. The authors agree on the benefits of using a scoping review to answer the research objective.

## Conclusions

Using a DLS in the later stages of dementia may differ from using it earlier in dementia. However, it can nevertheless compensate for weakening abilities in older adults. The results depict various ways to use a DLS to care for older adults with dementia. Although time constraints may complicate implementation, a DLS can be easily accessible. The content is in one place and available if the older adult should move to another residential care facility or be admitted to a hospital. The implications are expected to be that older adults with dementia can receive PCC through a DLS based on their wishes. However, more research is needed to understand how to increase the availability of DLSs for those who do not have relatives to assist in adding relevant material, for example, through interviews and surveys. A DLS can enable symmetric communication and serve as an intergenerational communication tool. It can be used for problem-solving and handling behavioural symptoms. A DLS should be based on the past, present, and future so that the older adult, their close relatives, and HCPs can create it, use it in nursing situations, and update it when necessary.

## Supplemental Material

sj-docx-1-dhj-10.1177_20552076241241231 - Supplemental material for The use of a digital life story to support person-centred care of older adults with dementia: A scoping review Supplemental material, sj-docx-1-dhj-10.1177_20552076241241231 for The use of a digital life story to support person-centred care of older adults with dementia: A scoping review by Helén Dellkvist, Ana Luiza Dallora, Line Christiansen and Lisa Skär in DIGITAL HEALTH

sj-docx-2-dhj-10.1177_20552076241241231 - Supplemental material for The use of a digital life story to support person-centred care of older adults with dementia: A scoping review Supplemental material, sj-docx-2-dhj-10.1177_20552076241241231 for The use of a digital life story to support person-centred care of older adults with dementia: A scoping review by Helén Dellkvist, Ana Luiza Dallora, Line Christiansen and Lisa Skär in DIGITAL HEALTH

sj-docx-3-dhj-10.1177_20552076241241231 - Supplemental material for The use of a digital life story to support person-centred care of older adults with dementia: A scoping review Supplemental material, sj-docx-3-dhj-10.1177_20552076241241231 for The use of a digital life story to support person-centred care of older adults with dementia: A scoping review by Helén Dellkvist, Ana Luiza Dallora, Line Christiansen and Lisa Skär in DIGITAL HEALTH
