# Supplementary material for: The use of a digital life story to support person-centred care of older adults with dementia: A scoping review
Source: Digit Health. 2024 Mar 20;10:20552076241241231. doi: 10.1177/20552076241241231 (PMC10953038; doi:10.1177/20552076241241231)
Supplement: sj-docx-1-dhj-10.1177_20552076241241231 - Supplemental material for The use of a digital life story to support person-centred care of older adults with dementia: A scoping review [file sj-docx-1-dhj-10.1177_20552076241241231.docx]

# Appendix 1: Final search terms

* Is used for truncation in the databases

| Main area | Cinahl | PubMed | Scopus | Google Scholar | Web of Science |
| --- | --- | --- | --- | --- | --- |
| Life story | Life Histories [MH], Autobiographies [MH], Life stor*, Life storybook*, Life history review* | Life histor*, Autobiograph*, Life stor*, Life storybook*, Life history review* | Life Histor*, Autobiograph*, Life stor*, Life storybook*, Life history review* | Life histor*, Autobiograph*, Life stor*, Life storybook*, Life history review*, | Life Histor*, Autobiograph*, Life stor*, Life storybook*, Life history review* |
| Digital health | Digital Health [MH], Digital | Digital Health, Digital | Digital Health, Digital | Digital Health, Digital | Digital Health, Digital |
| Welfare technology | Welfare technolog* | Welfare technolog*, | Welfare technolog* | Welfare technolog* | Welfare technolog* |
| Multimedia technology |  | Multimedia technolog* |  |  |  |
| Healthcare professionals |  |  |  | Healthcare professionals |  |
