# Supplementary material for: The use of a digital life story to support person-centred care of older adults with dementia: A scoping review
Source: Digit Health. 2024 Mar 20;10:20552076241241231. doi: 10.1177/20552076241241231 (PMC10953038; doi:10.1177/20552076241241231)
Supplement: sj-docx-2-dhj-10.1177_20552076241241231 - Supplemental material for The use of a digital life story to support person-centred care of older adults with dementia: A scoping review [file sj-docx-2-dhj-10.1177_20552076241241231.docx]

# Appendix 2: Presentation of database searches

## Searches in Cinahl

Searches were done in “all fields”. No filters were used. * is used for truncation.

| Date | Search string | Items found |
| --- | --- | --- |
| 2022-12-27 | (MH ”Digital Health+”) | 23079 |
| 2022-12-27 | (MH ”Digital Health+”) OR digital OR “welfare technolog*” | 77342 |
| 2022-12-27 | (MH "Life Histories") | 1385 |
| 2022-12-27 | (MH "Autobiographies") | 567 |
| 2022-12-27 | “life stor*” OR “life storybook*” OR “life history review*” | 2837 |
| 2022-12-27 | (MH "Life Histories") OR (MH "Autobiographies") OR “life stor*” OR “life storybook*” OR “life history review*” | 4646 |
| 2022-12-27 | (MH ”Digital Health+”) OR digital OR “welfare technolog*” AND (MH "Life Histories") OR (MH "Autobiographies") OR “life stor*” OR “life storybook*” OR “life history review*” | 43 |

## Searches in Scopus

Searches on social sciences and keywords regarding “autobiography” and “life history” were filtered. * is used for truncation.

| Date | Search string | Items found |
| --- | --- | --- |
| 2023-01-04 | ”digital health” OR digital OR “welfare technolog*” | 922494 |
| 2022-01-04 | "life histor*" OR "autobiograph*" OR "life stor*" OR "life storybook*" OR "life history review*" | 51177 |
| 2022-01-04 | "digital health" OR digital OR "welfare technolog*" AND "life histor*" OR "autobiograph*" OR "life stor*" OR "life storybook*" OR "life history review*" | 105 |

## Searches in Google Scholar

Searches were done in “all fields”. No filters were used. * is used for truncation.

| Date | Search string | Items found |
| --- | --- | --- |
| 2022-12-27 | ”digital health” OR digital OR “welfare technolog*” AND "life histor*" OR "autobiograph*" OR "life stor*" OR "life storybook*" OR "life history review*" | 1440 |
| 2022-12-27 | ”digital health” OR digital OR “welfare technolog*” AND "life histor*" OR "autobiograph*" OR "life stor*" OR "life storybook*" OR "life history review*" AND “healthcare professionals” | 39 |

## Searches in PubMed

* is used for truncation.

| Date | Search string | Items found |
| --- | --- | --- |
| 2023-01-09 | "Autobiography" [Publication Type] OR "Autobiographies as Topic"[Mesh] Sort by: Most Recent | 5,056 |
| 2023-01-09 | (((("life stor*") OR ("life histor*")) OR ("life story review*")) OR (autobiograph*)) OR ("life storybook*") | 36,944 |
| 2023-01-09 | ((((("life stor*") OR ("life histor*")) OR ("life story review*")) OR (autobiograph*)) OR ("life storybook*")) AND ("Autobiography" [Publication Type] OR "Autobiographies as Topic"[Mesh]) | 5,056 |
| 2023-01-09 | ((("digital health") OR (digital)) OR ("welfare technolog*")) OR ("multimedia technolog*") | 221,213 |
| 2023-01-09 | (((("digital health") OR (digital)) OR ("welfare technolog*")) OR ("multimedia technolog*")) AND (((((("life stor*") OR ("life histor*")) OR ("life story review*")) OR (autobiograph*)) OR ("life storybook*")) AND ("Autobiography" [Publication Type] OR "Autobiographies as Topic"[Mesh])) | 10 |

## Searches in Web of Science

Filters were used in the final search string in the table: Social Sciences Other Topics or Geriatrics Gerontology or Nursing or Health Care Sciences Services (Research Areas). * is used for truncation.

| Date | Search string | Items found |
| --- | --- | --- |
| 2023-01-03 | KP=("digital health") | 111 |
| 2023-01-03 | ALL=(digital)) AND ALL=("digital") | 924,204 |
| 2023-01-03 | ALL=("welfare technolog*") | 4,138 |
| 2023-01-03 | ALL=("life history") | 77,913 |
| 2023-01-03 | ALL=("autobiograph*") | 27,345 |
| 2023-01-03 | ALL=("life stor*") | 6,071 |
| 2023-01-03 | ALL=("life storybook*") | 7 |
| 2023-01-03 | ALL=("life history review*") | 9 |
| 2023-01-03 | KP=("digital health") OR ALL=(digital)) AND ALL=("digital") OR ALL=("welfare technolog*") | 928,241 |
| 2023-01-03 | ALL=("life history") OR ALL=("autobiograph*") OR ALL=("life stor*") OR ALL=("life storybook*") OR ALL=("life history review*") | 110,298 |
| 2023-01-03 | KP=("digital health") OR ALL=(digital)) AND ALL=("digital") OR ALL=("welfare technolog*") AND ALL=("life history") OR ALL=("autobiograph*") OR ALL=("life stor*") OR ALL=("life storybook*") OR ALL=("life history review*") | 661 |
| 2023-01-03 | KP=("digital health") OR ALL=(digital)) AND ALL=("digital") OR ALL=("welfare technolog*") AND ALL=("life history") OR ALL=("autobiograph*") OR ALL=("life stor*") OR ALL=("life storybook*") OR ALL=("life history review*") | 44 |
