# Supplementary material for: The use of a digital life story to support person-centred care of older adults with dementia: A scoping review
Source: Digit Health. 2024 Mar 20;10:20552076241241231. doi: 10.1177/20552076241241231 (PMC10953038; doi:10.1177/20552076241241231)
Supplement: sj-docx-3-dhj-10.1177_20552076241241231 - Supplemental material for The use of a digital life story to support person-centred care of older adults with dementia: A scoping review [file sj-docx-3-dhj-10.1177_20552076241241231.docx]

# Appendix 3: Charting of articles included in the qualitative content analysis

| Author, year, country, and type (a) | Title | Aim of the study | Design, method, context, and participants | Important findings |
| --- | --- | --- | --- | --- |
| Alm N, Astell A, Ellis M, et al.  2004  Scotland  Research article | A cognitive prosthesis and communication support for people with dementia | To develop a reminiscence system as a cognitive and communication aid. | In a pilot, a study assessment was made using a structured interview, and, for carers, a self-report questionnaire with a Lickert scale was used for responding. Three men and three women with dementia and six carers took part. Three people with dementia were seen at home with their family carer, and the other three were caught in daycare with a care staff member. | Computers can promote and maintain a conversation with people with dementia. A multimedia reminiscence system can assist people with dementia in talking about a wide range of topics. One positive benefit of the wide range of materials available is that care staff can use the system with little or no background preparation. |
| Astell A, Alm N, Gowans G, et al.  2008  UK  Long paper | Involving older people with dementia and their carers in designing computer-based support systems: some methodological considerations | To develop and evaluate a multimedia computer system to support communication between people with dementia and caregivers. |  | The intention was to develop a system for individual use, but there would be potential for developing a similar presentation for group enjoyment and comment. Using generic items and personal items is not necessary. |
| Astell A, Ellis M, Bernardi L, et al.  2010  Scotland  Research article | Using a touchscreen computer to support relationships between people with dementia and caregivers | To examine the interaction behaviour of care staff and people with a dementia diagnosis during reminiscing using both verbal and nonverbal measures. | A user-centred design. Eleven persons with dementia (six women) from daycare and residential facilities. Dyadic study sessions. | People with dementia can use the touch screen system, and the contents prompt them to reminisce. The system supports caregivers to interact with people with dementia as more equal participants in the conversation. Interacting with the touch screen system is engaging and enjoyable for people with dementia and caregivers alike and provides a supportive interaction environment that positively benefits their relationships. |
| Cohene T, Baecker R, Marziali E, et al.  2007  Canada  Research article | Memories of a life: A design case-study for Alzheimer’s disease | To design guidelines and enhancements for the main study. | Exploratory case study. Participatory design. Interaction with groups of individuals in moderated recreational therapy activities. The groups ranged from 5 to 35 mid and late-stage Alzheimer’s disease individuals and were moderated by healthcare staff and several volunteers. | People with dementia can interact with multimedia. Active participation is more stimulating than passive. Different stakeholders experience different effects. |
| Davis B and Shenk D  2015  USA  Research article | Beyond reminiscence: Using generic video to elicit conversational language | To measure engagement with persons with dementia that promotes talking. | Low-technology, homemade generic and personalised videos were tested with two samples of persons with dementia to increase engagement and support the retention of identity. Compare generic and personal videos based on photos. | The persons with dementia showed a slight preference for looking at personalised videos. They produced a more comprehensive range of conversational language topics and phrasal patterns in response to the generic videos. |
| Edling I and Söderquist Sandin N  2018  Sweden  Thesis | High-technological conversation support as a group activity for people with dementia - An analysis of interaction when using the CIRCA application | To promote social interaction for people with dementia through group discussion sessions using the Computer Interactive Reminiscence and Communication Aid (CIRCA). A further aim was to contribute knowledge about the potential of high-tech communication aids to facilitate and support communication in dementia. | The group sessions comprised seven hours of material  and 15 minutes of video recordings. Participants: Persons with dementia. | The sequences of conversations could touch on CIRCAS material, the handling of the tablet or created as a result of the material. Characteristic interaction phenomena were good group dynamics with laughter, humour, singing and social inclusion, with participants contributing information, personal experiences and questions to each other. An equal interaction between participants and between participants and the facilitator. Participants felt that the group sessions with CIRCA were good, fun, and educational, provided topics for discussion, and simplified the conversations compared to those without support. |
| Elfrink TR, Zuidema SU, Kunz M, et al.  2017  Netherlands  Research article | The effectiveness of creating an online life story book on persons with early dementia and their informal caregivers: a protocol of a randomized controlled trial | The main objective is to study the effects of the OLSB on neuropsychiatric symptoms. The study has four secondary objectives: 1) to study the effectiveness of the intervention on the burden and quality of life of the primary informal caregiver; 2) to provide a preliminary health-economic evaluation; 3) to study the (time to) nursing home admittance as a longer-term effect; 4) to provide a process evaluation. | A randomised controlled trial with individual randomisation to one of two conditions is conducted: 1) intervention “Online Life Story Book”; 2) control condition (care as usual). Participants are persons with early dementia and their primary caregivers. In the intervention OLSB, a trained volunteer guides the participants through creating a timeline of their lives during five meetings within 8-10 weeks. | The online life storybooks were primarily created in individual sessions focusing on persons with dementia and (in)formal caregivers. Showed the value of life storybooks in triggering memories and improving the relationship with the person with dementia. Effects on autobiographical memory, depression, quality of relationship with informal caregivers, the burden of informal caregivers and on attitudes and knowledge of formal caregivers. |
| Gowans G, Dye R, Campbell J, et al.  2004  Austria  Conference article | Designing a multimedia conversation aid for reminiscence therapy in dementia care environments | To demonstrate how this adaptable, expansive, immediate, and engaging tool can contribute significantly to the quality of life in dementia care units. | A user-centred design with interdisciplinary collaboration with persons with dementia and care staff. | CIRCA appears to promote a relaxed atmosphere. Prompted memories that none of the caregivers had heard before. |
| Hamel AV, Sims TL, Klassen D, et al.  2016  USA  Research article | Memory matters: A mixed-methods feasibility study of a mobile aid to stimulate reminiscence in individuals with memory loss | To test the feasibility and acceptability of a mobile tablet application to enhance reminiscence among persons living with dementia, called Memory Matters. | The feasibility of “Memory Matters” (MM), a mobile device application developed to promote reminiscence, was evaluated using a parallel convergent mixed-methods design. Eighteen persons with memory loss (PWML) and eight family members were enrolled on a long-term care facility and asked to use Memory Matters (MM) for four weeks. Participants were observed using MM at enrollment and two weeks and completed one-month interviews. Six staff participants completed a system review checklist and focus group at one month. | Participants reported real-time social engagement, ease of use, and other benefits. However, PWMLs were only able to overcome barriers with assistance. Empirical data indicated that family and staff perceived MM favourably. Participants agreed that MM could provide stimulating, reminiscence-based activity. |
| Hernández Maillard Y and Wahlqvist Å.  2020  Sweden  Thesis | Interaktion vid demens. Inverkan av digitala levnadsberättelser och kommunikationspartnerträning | The present study aimed to study the impact digital life stories, in the application CIRCUS, may have on interaction between nursing staff and a person with dementia (PWD) and the participants’ experiences of digital life stories. Further, the study aimed to investigate whether differences arose in the interaction after the nursing staff received communication partner training, where they got to observe themselves during a conversation with the PWD. | The study was conducted in an activity dementia centre where all participants were recruited; two nursing staff and two PWD. Participants were divided into two conversational pairs, and a total of four conversation sessions and eight interviews were recorded with video and audio. Before the second conversation session, the nursing staff participated in individual communication partner training and received general strategies for facilitating communication in dementia. | In summary, digital life stories positively affect communication with a PWD. Communication partner training also has a positive effect when communicating with a PWD. In conclusion, the present study shows that PWD is competent interlocutors and that adequate support from the interlocutor highlights this fact. |
| Maiden N, D’Souza S, Jones S, et al.  2013  UK  Discussion article | Computing technologies for reflective, creative care of people with dementia | To discuss mobile applications. | Discussion article | Mobile applications may support person-centred care and provide a social environment. Applications can contain personal information and ease communication with a person with dementia. |
| Mulvenna MD, Doyle L, Wright T, et al.  2017  UK  Conference article | Behavioural usage analysis of a reminiscing app for people living with dementia and their carers | The study aimed to examine older people's attitudes toward using a device to reminisce instead of a card-based approach. Another study objective was to see if the different types of images made a difference in recall. | Feasibility study. Participants were recruited from two locations in Northern Ireland to reflect a mix of urban and rural dwellers. There were 19 participants in total, with an average age of 71. Seven of the participants were male. A study control group used traditional photographs mounted on cards, while the device group used Apple® iPad® devices with touch-screen navigation of pictures. | Work-in-progress in a feasibility study of facilitated reminiscence for people with dementia, and results show promise in understanding the behaviour of interactive technologies. |
| O’Philbin L, Woods B, Windle G  2020  UK  Research article | Implementing digital life story work for people with dementia: Relevance of context to user experience | To explore user experiences of three different implementations of digital life story works. | Sixteen participants (six persons with dementia, six family caregivers and four care staff) working in a dementia care home took part. Participants were interviewed and participated in workshops. | Evidence for the feasibility and positive impact of a supported digital life story intervention and use of digital life storybooks in three contexts. Provides valuable insight and feedback for the future development and implementation of this approach in research and practice. |
| Samuelsson C and Ekström A  2019  Sweden  Research article | Digital communication support in interaction involving people with dementia | To further understand how digital communication support may be used in interactions involving people with dementia and to investigate how participants experience communication with and without technical aids. | The study was carried out in a Swedish context, and three dyads of older women with dementia and professional carers participated in the study. The dyads interact in the home environments of the persons with dementia using tablet computers and two web-based applications with generic pictures, videos, and music files (Computer Interactive Reminiscence and Communication Aid, CIRCA) and personalised pictures and films (Computer Interactive Reminiscence and Communication University of Sheffield, CIRCUS). The data include twenty-one video-recorded activities. | The applications support the dyads in finding things to talk about. The participants speak both about the material and the memories associated with the material. The participants experienced the use of communication aids as positive. |
| Subramaniam P and Woods B  2016  UK  Research article | Digital life storybooks for people with dementia living in care homes: an evaluation | To establish an evidence-base for the acceptability and efficiency of using multi-media digital life storybooks with people with dementia in care homes, in comparison with conventional life storybooks, considering the perspectives of people with dementia, their relatives and care staff. | Participatory design was used to create a life story movie based on a previously completed conventional life storybook with six older adults with dementia (four females. Relatives were involved in helping the participant to provide additional information and materials for the digital life storybook. Quantitative and qualitative approaches were used in this multiple-case study design. Questionnaires were developed and repeated for quantitative purposes four weeks after the life story movie was completed. Semistructured interview questions were designed to collect participant, relatives, and care staff feedback. | Five of the six participants showed additional improvement in quality of life and autobiographical memory measures. All participants showed improvement or stability in depression scores. Participants, relatives, and care home staff viewed digital life storybooks as helpful in triggering memories and largely positive emotions. Participants’ case vignettes were presented to document the impact of the digital life storybook. |
| Thoft DS, Möller AK, Möller AKK  2022  Denmark  Research article | Evaluating a digital life story app in a nursing home context – A qualitative study | To evaluate how digital life stories can be used to support person-centred care focusing on benefits and challenges experienced by the staff and management team in a nursing context. | Six healthcare professionals and two management team members at a nursing home were recruited. Participant observations with informal interviews of the formal health career were conducted in two-day shifts and two-night shifts. Field notes were taken during the observations. Also, individual semi-structured interviews were held with four of the recruited participants. This article adheres to the Standards for Reporting Qualitative Research (SRQR). | Person-centred care and life story work as interrelated approaches, pros and cons related to the My Life Story app, and how time and ICT can restrict life story work. |
| Upton D, Upton P, Jones T, et al.  2011  UK  A report commissioned by the Department of Health West Midlands | Evaluation of the impact of touch screen technology on people with dementia and their carers within care home settings | To assess the effectiveness and effects of touchscreen technology on the day-to-day lives of people with dementia and the staff engaged in their care. | Staff and residents’ experience of touchscreen technology was explored using a combination of topic-guided interviews, focus groups, case studies and field observations. | People with dementia and the staff involved in their care reported mainly positive experiences using touchscreen technology. The use of touchscreen devices such as the iPad in dementia care was found to have several advantages: Supporting reminiscence, aiding recall, increasing interpersonal interactions, intergenerational communication, staff-resident relationships, improving quality of life and ease of use. |
| Damianakis T, Crete-Nishihata M, Smith KL, et al.  2009  Canada  Research article | The psychosocial impacts of Multimedia Biographies on persons with cognitive impairments | To observe Alzheimer’s disease and mild cognitive impairment patients’ responses to personalised multimedia biographies. | An interdisciplinary team of multimedia biographers and social workers interviewed 12 family members of persons with AD and MCI. We filmed patients’ responses to watching the MBs and conducted follow-up interviews with the families and patients 3 and 6 months after the initial viewing. Qualitative analytic strategies extracted themes and critical issues identified in the filmed and interview response data. | Evoked long-term memories stimulated reminiscing, brought mostly joy but occasionally moments of sadness to the person with dementia, aided family members in remembering and better understanding their loved ones, and stimulated social interactions with family members and with formal caregivers. |
| Hashim A, Rias RM, Ismail AN, et al.  2015  Malaysia  Conference article | The development of an individualized digital memory book for Alzheimer’s disease patient: A case study | To present a personalised digital memory book combining reminiscence and cognitive stimulation therapy. | A case study. A 74-year-old woman tested an application. | The results indicated positive feedback and a good level of user satisfaction. The patient felt motivated and enjoyed using the application as it could support managing her daily activities, help her reminiscence, and improve her cognitive function. |
| Alm N, Dye R, Gowans G, et al.  2007  Scotland  Conference article | A communication support system for older people with dementia | To present a communication support system. | A multidisciplinary team of software engineers, psychologists, and designers developed CIRCA with input from potential users, their families, and professional caregivers throughout the design process. Eighteen people with dementia (13 women and five men) from several day-care and residential facilities participated in the evaluation: videotaped sessions and interviews. | The system showed itself superior to traditional methods of organising a reminiscence session. It helped the person with dementia take more control over the direction of the conversation and relieved the caregiver of the burden of responsibility for keeping the conversation going. |
| McAllister M, Dayton J, Oprescu F, et al.  2020  Australia  Research article | Memory Keeper: A prototype digital application to improve engagement with people with dementia in long-term care (innovative practice) | To provide more meaningful support to people with dementia. | Pilot study. The trial was conducted within the dementia wing of a long-term care facility. Participants in the trial were three residents with dementia and their six significant others. The Lifestyle Coordinator of the facility also participated. | Valuable in improving the quality of engagement and making visits more enjoyable. |
| Pekkarinen S, Melkas H, Kuosmanen P, et al.  2013  Finland  Research article | Towards a more social orientation in gerontechnology: Case study of the “reminiscence stick” | Through a case study, this article aims to investigate how social orientation can be realised in gerontechnology’s objectives. | A user-driven development process of a “mStick,” a tool for storing various biographical material: photographs, texts, audio and video clips, and using it in elderly care services. Qualitative data were collected in 11 pilot cases. | The mStick is prevention by offering meaningful content to life by utilising the older adult’s resources. It helps to see the person behind the disease. |
| Pang GHK and Kwong E  2015  Hongkong (a particular administrative region of China)  Conference article | Considerations and design on apps for elderly with mild-to-moderate dementia | To present the development of applications for people with dementia. | Presentation of applications that can support reminiscence | It may help the elder recall early memories; to support reminiscence. It can reduce anxiety and depression and bring out positive emotions. Provides quick and easy access to photos/videos of relatives and friends. The nurse or caretaker can know about the history and preferences of the elder and hence provide more understanding of the person. Better decision-making and tailored care can be given. |
| Wallace J, Thieme A, Wood G, et al.  2012  UK  Conference article | Enabling self, intimacy and a sense of home in dementia: An enquiry into design in a hospital setting | To describe an interactive art piece. | Two workshops with staff from a dementia unit in a hospital in the UK. The participants tested an interactive art piece. | The art piece served as a window on dementia and the institution, leading to several insights and implications for design. |
| Catala A, Nazareth D, Félix P, et al.  2020  Spain  Conference article | Emobook: A multimedia life story book app for reminiscence intervention | To present the design process and implementation of the app Emobook. | Usage tracking study. Meeting discussions, material enactments, mock-up screen design and interactions. A focus group was conducted with an occupational therapist, a social educator, a speech therapist who participated in the design process, and a student researching personal therapies. | The therapists confirm that the app fully supports individual reminiscence interventions. The app can help to define topics in a similar rationale and be a tool to initiate the story and stimulate conversations. |
| Samuelsson C  2020  Sweden  Web-based presentation | Digitala samtalsstöd vid demenssjukdom | To present digital conversation support for dementia. | Presenting research about CIRCA. | Can develop communicative networks. May promote health and memory support when using CIRCA. |
| Webster G and Hanson WL  2014,  Scotland  Research article | Technology for supporting care staff in residential homes | To evaluate computer vs paper presentation of biographical information in different use-case scenarios. | Description of the development of a software tool for use by care staff called Portrait and report about two studies related to factors affecting technology use by this population. The research participants were care staff from three care homes in Scotland. All of the participants had yet to experience Portrait. | Care managers and care staff positively received the design and may provide care staff with essential but limited personal and social information about residents. |
| Davison TE, Nayer K, Coxon S, et al.  2016  Australia  Research article | A personalised multimedia device to treat agitated behaviour and improve mood in people with dementia: A pilot study | To describe the results of a pilot trial of Memory Box as a treatment for anxiety, depression and agitated behaviours. | An eight-week randomised, single-blinded cross-over trial comparing Memory Box with a control condition that offered equivalent contact with research staff—a pilot study. Eleven nursing home residents with mild to severe dementia and daily agitated behaviours completed the study. | There were significant reductions in depressive and anxiety symptoms during the course of the intervention. The system shows promise as a tool to assist families and nursing home staff in improving the well-being of cognitively impaired older adults with agitated behaviours. |
| Bhar S, Stargatt J, Collins R, et al.  2022  Australia  Research article | Preliminary evidence for the impact of digital life stories about aged care residents on staff knowledge and understanding regarding those residents: A single arm trial | To examine the impact of digital stories about aged care residents on staff knowledge and understanding regarding those residents. | This study used a single-arm trial design. The study was conducted in four residential aged-care facilities. Short digital life stories (3–4 min) of eight residents were constructed by student volunteers over six months. Participants (n = 53 care staff) completed a self-report measure of their knowledge and understanding of a resident before and after watching the resident's story. The study adhered to guidelines for Strengthening the Reporting of Observational Studies in Epidemiology (STROBE) | Improvement of knowledge and understanding regarding residents. |
| Kikhia B, Hallberg J, Bengtsson J, et al.  2010  Sweden  Research article | Building digital life stories for memory support | To describe a hypothesis that memory entities are significant for building life logs. | Creation of digital life stories using life-logging entities with help from a memory support system. Sensor data, such as Bluetooth and GPS samples, and media, such as images, are collected automatically by devices that the person carries. | The proposed system enables real-time support and media-enriched life logs for integrated assistive services using established reminiscence processes and state-of-the-art technology. |
| Lynch S, Reilly E, Lowe F, et al.  2016  Ireland  Conference article | Creating life stories through digital technology as a means of supporting person-centred, relationship-based care across the continuum of dementia | To build life stories through digital technology to support adults with an intellectual disability across the continuum of dementia. | A multi-step approach was taken. First, a comprehensive survey in an easy-to-read format was distributed throughout the Dublin Service of the Daughters of Charity Disability Support Service to gather information and gauge interest among 380 people aged 40 years and over. A sub-cohort of people with Down syndrome who are at increased risk of dementia and had a diagnosis of dementia were included. Next, an exploration was undertaken of currently available Life Story and music apps and a suite of easy-to-use apps was identified and then pilot-tested. | Digital life stories are a compelling means of facilitating meaningful communication and supporting relationship-based, person-centred care across the continuum of dementia. They are very acceptable to the individual, family, and staff carers. Structured training, easy-to-read support material, and organisational commitment are critical for sustainability. |
